# Genomic insertion of lentiviral DNA circles directed by the yeast Flp recombinase

**DOI:** 10.1186/1472-6750-8-60

**Published:** 2008-08-09

**Authors:** Brian Moldt, Nicklas H Staunstrup, Maria Jakobsen, Rafael J Yáñez-Muñoz, Jacob G Mikkelsen

**Affiliations:** 1 Department of Human Genetics, University of Aarhus, Aarhus, Denmark; 2 School of Biological Sciences, Royal Holloway-University of London, Egham, Surrey, UK

## Abstract

**Background:**

Circular forms of viral genomic DNA are generated during infection of cells with retroviruses like HIV-1. Such circles are unable to replicate and are eventually lost as a result of cell division, lending support to the prevalent notion that episomal retroviral DNA forms are dead-end products of reverse transcription.

**Results:**

We demonstrate that circular DNA generated during transduction with HIV-1-based lentiviral vectors can be utilized as substrate for gene insertion directed by nonviral recombinases co-expressed in the target cells. By packaging of lentiviral genomic RNA in integrase-defective lentiviral vectors, harboring an inactive form of the viral integrase, the normal pathway for viral integration is blocked and circular vector DNA accumulates in transduced cells as a result. We find that the amount of DNA circles is increased 4-fold in cells transduced with integration-defective vectors relative to cells treated with integrase-proficient vectors. By transduction of target cells harboring engineered recognition sites for the yeast Flp recombinase with integration-defective lentiviral vectors containing an ATG-deficient hygromycin B selection gene we demonstrate precise integration of lentiviral vector-derived DNA circles in a drug-selective approach. Moreover, it is demonstrated that *trans*-acting Flp recombinase can be delivered by Flp-encoding transfected plasmid DNA or, alternatively, by co-transduced integrase-defective lentiviral vectors carrying a Flp expression cassette.

**Conclusion:**

Our data provide proof-of-principle that nonviral recombinases, like Flp, produced by plasmid DNA or non-integrating lentiviral vectors can gain access to circular viral recombination substrates and facilitate site-directed genomic insertion of such episomal DNA forms. Replacement of the normal viral integration machinery with nonviral mediators of integration represents a new platform for creation of lentiviral vectors with an altered integration profile.

## Background

Integration of foreign DNA plays a pivotal role in genetic engineering, animal transgenesis, and therapeutic gene transfer. Lentiviral vectors (LV) efficiently insert genetic cargo in both dividing and non-dividing cells and have attracted, therefore, major attention as a gene transfer tool. During lentiviral transduction, linear vector DNA is associated with viral and cellular proteins in the preintegration complex (PIC) which is transported across the nuclear membrane and facilitates tethering of viral genomic DNA to chromatin. As part of the PIC, cellular LEDGF/p75 is thought to anchor viral DNA on chromatin, allowing the viral integrase to insert viral DNA in a fashion that supposedly favors integration in actively transcribed genes [1-3]. Great interest is attracted to ways of altering the lentiviral integration profile, allowing gene insertion in predetermined and safe vector landing sites. It remains unknown, however, whether lentiviral integration can be directed by alternative integration machineries, despite the integrity of the PIC and the exquisite involvement of cellular factors in nuclear entry and chromatin association.

During lentivirus infection circular forms of the viral genomic DNA are generated by either non-homologous end joining (NHEJ) of the full-length linear viral DNA (creating 2-LTR circles) [4], or by homologous recombination between the two LTRs of the episomal viral DNA (creating 1-LTR circles) [5]. Such episomal DNA circles have traditionally been considered dead-end products of reverse transcription [5] and are eventually lost together with episomal linear vector forms as a result of host cell division. However, integration-defective lentiviral (IDLV) vectors carrying an inactive integrase protein that abolish the normal viral integration pathway have recently emerged as novel efficient gene carriers, facilitating high levels of transient expression from linear and circular DNA forms [6-9]. Increased persistence of transgene expression in dividing cells has been demonstrated by allowing circles harboring the simian virus 40 (SV40) origin of replication to replicate episomally in cells containing the SV40 large T antigen [9]. Moreover, non-integrating lentiviral vectors have been shown to facilitate stable *in vivo *therapeutic levels of transgene expression in non-dividing neuronal cells [8] and muscle [6]. Recently, the episomal nature of the integration-defective vectors was further exploited as a template source for high-efficient gene correction by homologous recombination in human cell lines and in human embryonic stem cells [10,11].

High stability and accessibility of DNA circles by cellular proteins have led us to suggest that stable circular DNA intermediates can be engineered to act as putative substrates for gene insertion by nonviral gene-inserting proteins. We test this hypothesis here by using a model system based on the actions of the site-directed Flp recombinase and demonstrate for the first time that LV-derived DNA circles can serve as a substrate for gene insertion facilitated by exogenous nonviral recombinases. Our findings provide evidence that *trans*-acting integrases are able to gain access to and insert transduced lentiviral DNA with possible application in (i) viral vector manipulation for improved viral gene transfer and (ii) Flp-based cell engineering methods focusing on hard-to-transfect cells and/or creation of site-directed gene insertions that do not contain bacterial remnants of plasmid DNA.

## Methods

### Vector construction

The HIV-1-derived Flp substrate vector, pLV/FRT-hygro, was generated by replacing the eGFP gene (BamHI/XhoI digestion) of the third generation SIN-vector pCCL.WPS.PGK-eGFP.WHV (a kind gift from Dr. Patrick Aebischer, Swiss Federal Institute of Technology, EPFL, Lausanne, Switzerland) with the puromycin resistance gene, PCR-amplified from pT/PGK-Puro [12] (creating pCCL.WPS.PGK-Puro.WHV), followed by insertion of the ATG-deficient FRT-hygro fusion gene (PCR-amplified from pcDNA5/FRT, Invitrogen) into the HpaI site located upstream of the cPPT The SB-based docking vector, pSBT/RSV-FGIP, was generated by amplifying the eGFP sequence (from peGFP.N1, Clontech) using a forward primer containing the 48-bp FRT sequence and inserting the resulting FRT.GFP fusion gene into MluI/XmaI-digested pSBT/RSV-hAAT [12] (generating pSBT/RSV-FRT.GFP) prior to insertion of an IRES-puro cassette (PCR-amplified from pecoenv-IRES-puro, kindly provided by Dr. Finn Skou Pedersen, University of Aarhus, Denmark), into the XmaI site. pLV/PGK-Flp was generated by replacing the eGFP gene of pCCL.WPS.PGK-eGFP.WHV with the Flpx9 gene [13] PCR-amplified from pCMV-Flp (obtained from A. Francis Stewart, University of California San Francisco, USA), the latter which contains the enhanced x9 Flp variant driven by a cytomegalovirus (CMV) promoter. pCMV-SB contains the SB10 transposase gene driven by a CMV promoter and has been described previously [14].

### Lentiviral vector production

HEK-293 and 293T cells were cultured at 37°C in 5% (v/v) CO_2 _and maintained in Dulbecco's modified Eagle's medium (Cambrex, Verviers, Belgium) with D-glucose (4.500 mg/liter) supplemented with 10% fetal calf serum, penicillin (100 U/ml), streptomycin (0.1 mg/ml), and L-glutamine (265 mg/liter). When selection was applied, puromycin (Sigma, St. Louis, MO) or hygromycin B (Invitrogen, Carlsbad, CA) was added to the growth medium to a final concentration of 1 μg/ml or 200 μg/ml, respectively.

VSV-G-pseudotyped lentiviral vectors were produced by CaPO_4_-transfection of 293T cells (seeded at 3 × 10^6 ^cells/dish in 10-cm dishes) with 3 μg pRSV-Rev, 3.75 μg pMD.2G (VSV-G), 13 μg pMDLg/pRRE (or 13 μg pMDLg/pRREintD64V [8] for production of integration-defective vectors) and 13 μg lentiviral vector plasmid. The supernatant was harvested two days post-transfection and polybrene was added to a final concentration of 8 μg/ml prior to transfer to target cells.

### Generation and transfection of FRT-tagged cell lines

FRT-tagged HEK-293-derived cell lines were generated by transfecting HEK-293 cells (seeded at 2 × 10^5 ^cells/well in 6 well plates) with 1.5 μg pSBT/RSV-FGIP and 0.5 μg pCMV-SB [14] (using 4 μl Fugene-6; Roche, Basel, Switzerland) and selecting for puromycin resistance. Resistant clones were isolated and expanded. For evaluating the efficacy of Flp-mediated insertion of transgenes into the engineered FRT docking site, FRT-tagged cell lines (seeded at 7 × 10^5 ^cells/dish in 10-cm dishes, n = 3) were CaPO_4_-transfected with 2 μg pLV/FRT-hygro and 10 μg pCMV-Flp or 10 μg pUC19 (negative control). Two days after transfection the cell lines were split, diluted, and selected for 10 days with hygromycin B prior to counting of hygromycin B-resistant colonies.

### Quantification of viral DNA forms in transduced cells

HEK/FGIP1 cells (seeded at 3 × 10^6 ^cells/dish in 10-cm dishes, n = 3) were transduced with LV/FRT-hygro or IDLV/FRT-hygro (~4.33 × 10^6 ^and ~3.79 × 10^6 ^pg p24, respectively). Hirt DNA was harvested 24 hours post-transduction. The amount of total HIV DNA and 2-LTR circles was quantified by real time PCR performed on an iCycler Thermal Cycler (Bio-Rad) using the DyNAmo HS SYBR Green qPCR Kit (Finnzymes, Espoo, Finland). Total HIV DNA was quantified by amplifying a part of the Woodchuck Hepatitis Virus (WHV) posttranscriptional regulatory element present in the vector backbone, and 2-LTR circles were amplified by using primers spanning the LTR-LTR junction. Copy numbers of total HIV DNA and 2-LTR circles were determined from standard curves created by PCR amplification on matching control DNA templates. Concentrations of p24 Gag were measured using a HIV-1 p24 ELISA kit (ZeptoMetrix, Buffalo, NY). To rule out possible overestimation of the total vector DNA due to putative contamination with plasmid DNA, we carried out a quantitative PCR analysis using a primer set amplifying an amplicon within the Amp resistance gene in the plasmid backbone. A pLV/FRT-hygro plasmid dilution series was used as qPCR standard to verify sufficient sensitivity of the assay to detect plasmid contamination. Contaminating plasmid DNA was detected within the range of the assay but was neglectable relative to levels of total vector DNA in transduced cells.

### Flp-directed insertion of lentiviral DNA circles

HEK/FGIP1 (n = 3) and HEK/FGIP2-6 (n = 1) cell lines were seeded at a total of 7 × 10^5 ^cells/dish in 10-cm dishes. For combined transfection-transduction assays the transfections were performed by CaPO_4 _using a total of 10 μg pCMV-Flp or 2 μg pLV/FRT-hygro, respectively. Transductions were performed using IDLV/FRT-hygro (~4.8 × 10^5 ^pg p24) or IDLV/PGK-Flp (~17 × 10^5 ^pg p24) vectors. The cells were grown in non-selective medium for two days before being subjected to hygromycin B selection. In co-transduction assays the cells were infected with IDLV/FRT-hygro (~4.8 × 10^5 ^pg p24) and IDLV/PGK-Flp (~17 × 10^5 ^pg p24) before being subjected to hygromycin B selection. All experiments were performed using an empty Flp-deficient vector as a negative control.

### Analyses of clones carrying inserted DNA circles

Southern blot analysis was performed using 10 μg XbaI-digested genomic DNA isolated from hygromycin B-resistant clones. The digested DNA was separated on a 0.8% agarose gel, transferred to a nitrocellulose membrane, hybridized to a 700-bp ^32^P-labeled probe, derived from the hygromycin B resistance gene, and subjected to autoradiography. HEK-293 genomic DNA spiked with pLV/FRT-hygro and digested with XbaI (which cuts at a single site in pLV/FRT-hygro) was utilized as a positive control. PCR-based analyses of Flp-directed circle insertions were performed using genomic DNA from isolated hygromycin B-resistant clones. Primer sequences are available upon request.

## Results and Discussion

**Figure 1:**
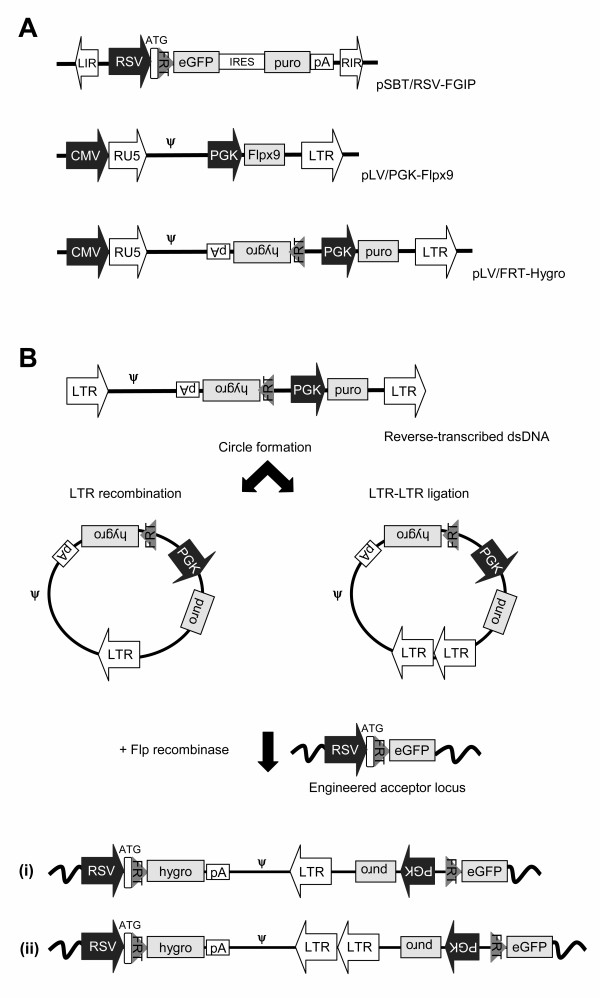
Strategy for Flp-directed integration of lentiviral DNA circles. (**A) **Schematic representation of vectors utilized for lentiviral transduction and generation of Sleeping Beauty vector-tagged cell lines. The lentiviral vector, pLV/FRT-hygro, is a third generation SIN vector containing an ATG-deficient FRT-hygro fusion gene located between the packaging signal (ψ) and the central polypurine tract (cPPT, not shown) flanked downstream by a selectable marker expression cassette. In the lentiviral SIN vector pLV/PGK-puro the PGK-driven Flpx9 expression cassette is located downstream from ψ and cPPT sequence (the latter not shown). The SB transposon docking vector, pSBT/RSV-FGIP, contains the left and inverted repeats of SB (LIR and RIR, represented by white arrows) and an expression cassette containing the RSV promoter driving a fusion gene consisting of an ATG-FRT-tagged eGFP gene, an IRES element, the puromycin-resistance gene, and a polyadenylation signal. (**B**) Schematic representation of site-directed integration of LV DNA circles. After reverse transcription of the viral RNA, circular forms of the viral genomic DNA are generated by either non-homologous end joining (2-LTR) or homologous recombination (1-LTR). These circles are normally considered dead-end products of reverse transcription but the FRT site will enable DNA circles to become substrates for Flp recombination. In cells harboring an engineered FRT site flanked by a promoter and a start codon, Flp-mediated insertion of the virus-derived (i) 1-LTR circles and (ii) 2-LTR circles will generate a functional hygromycin B resistance expression cassette. To block the normal viral integration machinery, integration-defective lentiviral vectors (IDLVs), carrying an inactive viral integrase protein (harboring the D64A mutation), were utilized as carriers of viral RNA. Puro, puromycin resistance gene; LTR, long terminal repeat; FRT, Flp recombination target site.

To provide proof-of-principle that *trans*-acting nonviral recombinases are able to gain access to lentivirally delivered substrates and facilitate their insertion, we designed an LV-derived hybrid integration system based upon the integrating properties of the yeast Flp recombinase. First, we constructed an LV substrate vector, pLV/FRT-hygro, containing an ATG-deficient FRT-hygro fusion cassette in the context of a self-inactivating (SIN) LV vector (Figure 1A). The Flp recombination target (FRT) sequence is recognized by the Flp recombinase which mediates recombination between two identical FRT sites. By including the FRT sequence in this vector, we reasoned that LV DNA circles generated during vector transduction would serve as substrates for Flp-dependent site-directed insertion of viral DNA into FRT sites engineered into the genome of transduced cells. Integration of the ATG-deficient FRT-hygro fusion gene into an engineered FRT site flanked upstream by a promoter and an ATG sequence would regenerate a functional hygromycin B expression cassette, allowing selection of cells containing site-directed vector insertion (Figure 1B). Hence, by using a site-directed Flp-based approach, we were able to score circle insertions selectively but also suspected a low circle insertion rate due to strong limitations on available target sites in the transduced cells.

**Figure 2:**
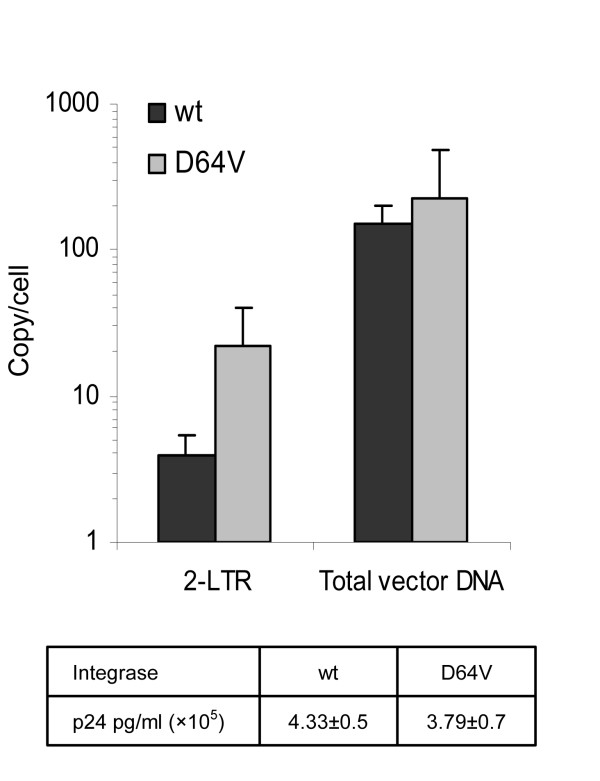
Blockage of the normal viral integration machinery increases the formation of LV DNA circles in transduced cells. HEK-SB/FGIP cells were infected with integration-proficient or integration-deficient lentiviral vectors. Hirt DNA was harvested 24 hours post-transduction and subsequently used for Q-PCR analysis to determine the amount of linear and circular lentiviral DNA. Transductions were performed in triplicates and presented as mean value + SD.

The conditions for the Flp-based integration machinery were optimized by packaging vector RNA in virus particles carrying an inactive viral integrase protein harboring the class I D64V mutation [15]. By blocking the normal viral integration machinery, we were able to increase the copy number of 2-LTR circles from 3.8 to 22 circles per cell (Figure 2) corresponding to a 3.9-fold increase of circular DNA (correlated to the total amount of HIV-1 DNA) available for Flp-based recombination in transduced cells (Figure 2). To rule out possible overestimation of the total vector DNA due to plasmid contamination, we carried out a quantitative PCR analysis using a plasmid-specific primer set amplifying an amplicon within the Amp resistance gene in the plasmid backbone. Although within the detection limit of the assay, the level of contaminating plasmids was neglectable relative to measured copy numbers of total vector DNA (data not shown) and considered, therefore, to be without notable influence on measurements of total vector DNA. Importantly, the integrase mutation did not influence production or transduction capacity of the viral particles, since p24 values of the virus preparations and the total amount of episomal vector DNA present in the transduced cells were comparable between vector preparations carrying active and inactive viral integrase (Figure 2). In accordance with previous reports [6,16] the titer of integrase-deficient vectors was reduced approximately 1000-fold (from ~10^8 ^to ~10^5 ^CFU/ml) compared to vectors carrying the active integrase (data not shown).

**Figure 3:**
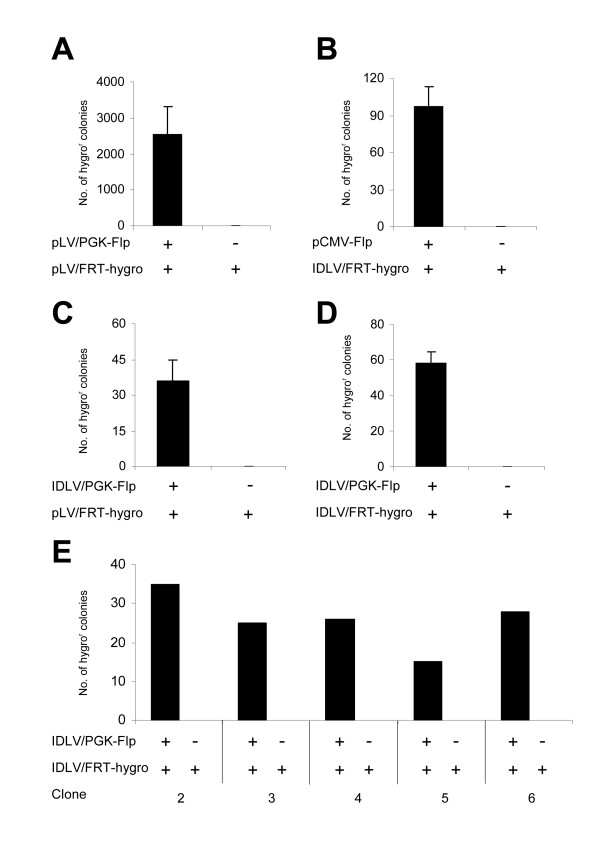
Episomal lentiviral DNA can act both as a substrate for Flp recombination and as a source of Flp recombinase for site-directed genomic integration. (**A**) Flp-mediated integration of plasmids into FRT-tagged HEK-derived cell line. The HEK/FGIP1 cell line was transfected with pLV/FRT-hygro and pCMV-Flp or a negative control and selected for hygromycin B resistance. (**B**) Flp-mediated integration of lentiviral DNA circles. HEK/FGIP1 cells were transfected with pCMV-Flp or a negative control one day prior to transduction with the IDLV/FRT-hygro vector. (**C**) Transient expression of Flp recombinase from integration-defective LV vectors is sufficient for site-directed insertion of donor plasmid. HEK/FGIP1 cells were transduced with IDLV/PGK-Flp or a negative control and on the following day transfected with pLV/FRT-hygro. (**D-E**) IDLV-encoded Flp catalyzes site-directed genomic integration of lentiviral DNA circles. HEK/FGIP1-6 cells were co-transduced with IDLV/FRT-hygro and IDLV/PGK-Flp or a negative control. Transfections/transductions were performed in triplicates (except for in panel E in which n = 1) and presented as mean value + SD.

To tag chromosomal DNA with recognition sequences for the Flp recombinase, we generated a Sleeping Beauty [14] (SB) transposon-derived docking vector, pSBT/RSV-FGIP, containing the Flp recombination target (FRT) sequence as part of a new active fusion variant of the eGFP reporter gene (Figure 1A). The eGFP fusion gene, containing an FRT sequence between the start codon and the remaining part of the eGFP coding sequence, was flanked downstream by an IRES element and the puromycin resistance gene. The docking SB transposon was inserted into the genomic DNA of HEK-293 cells by co-transfection of pSBT/RSV-FGIP with pCMV-SB, a plasmid encoding the SB transposase. To provide optimal conditions for subsequent Flp-based gene insertion, we utilized conditions which in our hands consistently allow insertion of >1 vector in transfected HEK-293 cells. The rationale was to create a cell line tagged with at least two docking sites. Among several eGFP-positive HEK-293 cell lines that were generated by puromycin selection, one cell line, HEK/FGIP1, was utilized for further studies. This particular cell line was found to contain at least 3 vector insertions, as determined by Southern blot analysis (data not shown). To confirm functionality of the FRT sequence in the context of the eGFP fusion gene, HEK/FGIP1 cells were co-transfected with 2 μg plasmid carrying an ATG-deficient FRT-hygro cassette (pLV/FRT-hygro) and 10 μg plasmid encoding Flp recombinase. Subsequent hygromycin B selection demonstrated efficient insertion into the engineered FRT site (Figure 3A). Analysis of the transfected HEK-SB/FGIP1 cells by quantitative PCR (data not shown) showed that each cell on average contained 1.3 × 10^4 ^± 2.3 × 10^3 ^FRT-tagged donor plasmids 24 hours after transfection, leading to an estimated insertion rate of 2.8 × 10^-7 ^Flp-based insertions per single copy of transfected FRT-hygro-containing plasmid in the culture. Notably, co-transfection of FRT-hygro substrate plasmid with a negative control plasmid did not result in colony formation, demonstrating the absolute lack of false positives in the system. As expected by the presence of >1 docking insertion in this cell line, eGFP expression was not turned off in hygromycin B resistant cells due to the fact that not all docking sites had been targeted by Flp recombination. However, subsequent PCR and sequence analysis of multiple hygromycin B-resistant clones confirmed correct Flp-based plasmid insertion in the eGFP gene of the integrated docking vector (data not shown). In summary, these data provide evidence that the Flp docking construction containing the novel FRT-eGFP fusion gene is functional in context of an integrated SB vector.

During lentiviral transduction double-stranded viral DNA is transported through the nuclear membrane as part of the PIC consisting of both viral and cellular proteins. Attempts to access the viral DNA by nonviral recombinases may therefore be hampered by the reduced DNA accessibility within the context of the PIC. To demonstrate that Flp indeed can gain access to the circular viral DNA and facilitate genomic insertion of LV circular substrates, we transfected the HEK/FGIP1 cell line with a Flp expression plasmid one day prior to transduction with the integration-defective (ID) LV/FRT-hygro vector (see Figure 1A). After hygromycin B selection 97 ± 15 resistant colonies were obtained (Figure 3B), whereas resistant colonies could not be detected in cells transfected with an empty control plasmid prior to IDLV/FRT-hygro transduction.

To examine whether IDLVs, co-transduced with DNA circle donor IDLVs, could serve as a source of Flp recombinase we created an LV SIN vector, pLV/PGK-Flpx9, containing a PGK-driven Flp gene (Figure 1A). First, HEK/FGIP1 cells were transduced with IDLV/PGK-Flp and on the following day transfected with an FRT-tagged plasmid substrate (pLV/FRT-hygro). After two weeks of hygromycin B selection, we detected 35 ± 9 drug-resistant colonies as a result of Flp-mediated plasmid insertion (Figure 3C), whereas transduction with a viral vector, IDLV/PGK-eGFP, lacking the Flp expression cassette did not result in any colony formation. These findings demonstrated that Flp recombinase, transiently expressed from integration-defective LV vectors, was able to confer substrate recombination and site-specific gene insertion. We finally co-transduced the HEK/FGIP1 cell line with IDLV/FRT-hygro and IDLV/PGK-Flp and subsequently selected transduced cells for hygromycin B resistance. In this setup, we obtained 58 ± 6 colonies per co-transduction (Figure 3D). To examine whether the efficiency of Flp-directed gene insertion varied among clones tagged with the docking vector, we generated five additional FRT-tagged HEK clones (HEK/FGIP2-6). All these cell lines demonstrated levels of eGFP expression that were comparable with the expression of eGFP in HEK/FGIP1 as measured by FACS analysis (data not shown). By co-transduction of these cell lines with IDLV/FRT-hygro and IDLV/PGK-Flp we obtained from 15 to 35 hygromycin B-resistant colonies (Figure 3E), indicating that efficiency of LV circle insertion varied only to a small degree between the tested FRT-tagged clones.

**Figure 4:**
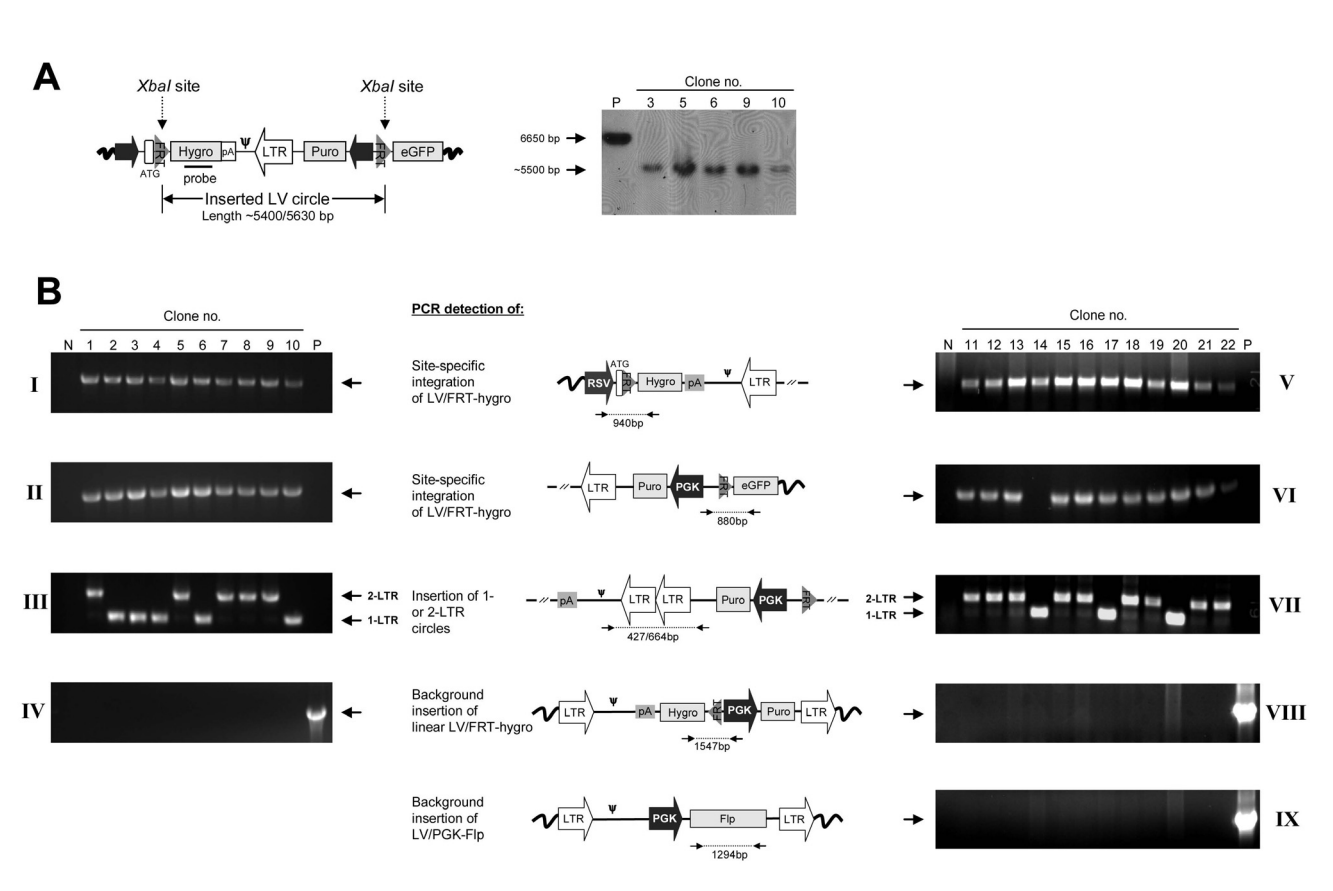
**Southern blot and PCR analysis confirm precise insertion of lentiviral derived DNA circles **(**A**) Southern blot analysis of representative hygromycin B-resistant clones containing inserted lentiviral circles. Genomic DNA was digested with Xba*I*, which cuts within the duplicated FRT sites, generating a ~5400/5630-bp fragment (1-LTR and 2-LTR insertions, respectively) recognized by the indicated hygro probe. Length differences between 1- and 2-LTR insertions could be resolved on a shorter exposure of the membrane (not shown). (**B**) PCR-based characterization of vector insertions in transduced cells. Genomic DNA from ten (taken from (**3B**)) and twelve (taken from (**3D**)) hygromycin B-resistant clones was used for PCR analysis to confirm site-specific insertion into an engineered FRT site (panel I–II and V–VI), integration of circular DNA substrates containing one or two LTRs (panel III and VII), and finally to verify that unspecific insertions of IDLV/FRT-hygro or IDLV/PGK-Flp had not occurred (panel IV and VIII–IX). N and P indicate negative (HEK/FGIP1) and positive (pLV/FRT-hygro or pLV/PGK-Flp) control clones, respectively.

To demonstrate that the DNA circles were precisely inserted by Flp into the FRT docking site, we first analyzed five representative clones by southern blot analysis of genomic DNA digested with Xba*I*, which cuts within the duplicated FRT sequences (Figure 4A). This analysis verified that full-length circles had been site-specifically inserted. To further characterize the inserted DNA circles 22 clones (10 and 12 clones from figure 3B and 3D, respectively) were isolated and analyzed. PCR amplification of genomic DNA using primer sets flanking the upstream and downstream FRT junction sites, respectively, resulted in PCR fragments indicative of site-specific circle insertion into the SB-tagged locus (Figure 4B, **panel I–II and V–VI**). Sequencing of these PCR products confirmed precise recombination between the genomic FRT site and the circle-associated FRT site, except for one clone (clone 14) in which part of the downstream region of the vector had been deleted. Although this clone carried marks of an inaccurate circle insertion, it could alternatively be the result of Flp-based recombination between a linear substrate and the genomic FRT site. In theory, linear substrates may be as efficient substrates as their circular counterparts but the outcome of vector incorporation into the genomic FRT site may differ. Whereas circular forms are resolved by the Flp recombinase into the genomic site without generating DNA breaks, linear forms containing free LTR ends would be expected to generate potentially harmful double-strand DNA breaks. Such complications would suggest that LV hybrid systems based upon cut-and-paste transposases acting on circular and linear substrates may be advantageous and perhaps safer than recombinase-based systems.

Next, we carried out PCR amplification and sequencing of fragments containing the LTRs of the site-directed insertions. Eight 1-LTR and fourteen 2-LTR circle integrations, created by Flp-based insertion of circles generated by homologous recombination and NHEJ, respectively, were detected (Figure 4B, **panel III and VII**) confirming that the integrated DNA in each clone was indeed derived from circular substrates. We analyzed 33 additional clones and from a total of 55 clones, 30 1-LTR and 25 2-LTR insertions were identified (data not shown). According to previous findings claiming a 9:1 ratio of 1-LTR to 2-LTR circles present in transduced cells [17], there appears to be a preference for insertion of 2-LTR circles in our system. Although this finding could reflect differences in the cellular location of circle species and/or their accessibility for proteins *in trans*, possible explanations remain speculative. Altogether, our data demonstrate that DNA circles generated during lentiviral transduction are indeed accessible for nonviral integrases *in trans *and may serve as substrates for genomic recombination by Flp protein transiently delivered by an integration-defective vector.

Based upon 2-LTR copy numbers, measured by q-PCR, we calculated an estimated insertion rate of 1.7 × 10^-5 ^Flp-based insertions per single copy of transduced 2-LTR circle (number of drug-resistant colonies containing a 2-LTR insertion/(number of 2-LTR circles per cell × total number of transduced cells)), leading us to suggest that 2-LTR circles, in comparison to transfected plasmid DNA, might be more efficient substrates for Flp-based recombination.

The D64V mutation in the HIV-1 integrase protein removes all enzymatic activity of the protein and has been reported to reduce integration of vector DNA 1.000–10.000-fold [6,15,16]. We reproducibly measured a 1000-fold reduction by colony-forming assays and, hence, could not formally rule out that integrase-independent insertion of linear IDLV/FRT-hygro or IDLV/PGK-Flp could have occurred in hygromycin B-selected clones. We therefore verified by PCR analysis that unspecific integration of linear vector DNA had not occurred in any of the hygromycin B-resistant clones (Figure 4B, **IV **and **VII–IX**), providing proof that the cell clones carrying a site-directed insertion did not carry additional background vector insertions.

Our findings provide, to our knowledge, the first proof-of-principle that integration of lentiviral DNA can be facilitated by a nonviral recombinase, thereby altering the integration profile of LV vectors – in this case towards a site-directed profile using DNA circles as a substrate for gene insertion. The normal catalytic activities of the lentiviral integrase were replaced in a drug-selective approach with the site-directed properties of the yeast Flp recombinase. As Flp recognition sites are not present in the human genome, an eGFP fusion gene containing the FRT sequences was inserted by a novel transposon-based FRT docking vector prior to IDLV transduction. The site-directed approach allowing insertion only in the genomic engineered sites resulted, as expected, in a fairly low insertion rate. As more potent alternatives, LV-hybrids with integration machineries derived from the phage ΦC31 integrase [18], hyperactive Sleeping Beauty transposases [19] or the AAV Rep protein [20] will have direct relevance in human cells, although cut-and-paste transposon systems would be preferred to mimimize the risk of generating double-strand DNA breaks as a possible outcome of recombinase-based insertion of linear substrates. Site-directed integration technologies based on the recombination capabilities of tyrosine recombinases as Cre or Flp have been widely used to direct targeted insertion of nonviral DNA plasmid substrates into genomic recognition sites. Flp-mediated plasmid insertion routinely allows genetic engineering of easy-to-transfect cell lines and has become pivotal in comparative gene expression studies in which integrated transgenes of interest may otherwise be variably influenced by the surrounding DNA. Our data describe a lentivirus-based technology with possible implication for Flp-mediated gene insertion in hard-to-transfect cell lines or potentially in FRT-tagged tissues or primary cells that are not easily transfected. Recent evidence has supported the fact that episomal lentiviral circles are highly stable in non-dividing cells and may provide persistent transgene expression in vivo [8]. For many applications, the stability of lentiviral circles in non-dividing cells does not call for genomic integration of the transgene. Nevertheless, stable viral episomes may represent permanent targets for recombinase-directed insertion of lentiviral circles in non-dividing cells.

The obvious difference in efficiency (as measured by the number of hygromycin B-resistant colonies) between plasmid- and the LV circle-based Flp integration systems (Figure 3A versus Figure 3B and 3D) may reflect differences in the availability of circular substrates in the two systems (plasmid DNA versus LV circles) or, alternatively, that plasmid DNA is somehow better suited for Flp-based insertion. Quantitative PCR analyses comparing the number of plasmids in transfected cells with the number of episomal 2-LTR DNA circles in LV-transduced cells indicated that about 600 times more episomal DNA circles are available in the plasmid-based system compared to the LV-based system (1.3 × 10^4 ^plasmids per cell compared to 22 2-LTR DNA circles per cell). However, as we found that 50% of the hygromycin B-resistant clones were the result of 2-LTR circle insertions, we estimate that the high number of plasmids available in transfected cells produced only 52 times more hygromycin B-resistant colonies than transduced 2-LTR episomes (2546 versus 48.5 colonies), leading to the assumption that LV-derived 2-LTR circular DNA is more efficiently integrated than plasmid-based substrates. Possible explanations for this finding, including varying substrate concentrations in different cellular compartments and differences in accessibility caused by structural substrate constraints (relaxed versus supercoiled substrates), are currently subjects for further scrutiny. Comparable levels of colony formation in experiments using IDLVs as a source of Flp recombinase and/or circular DNA substrates (Figure 3B, C, and 3D) indicate that both virus-derived Flp and DNA substrates are limiting factors for efficient Flp-based gene insertion.

By integrating viral circles rather than plasmid DNA, we obtained Flp-based insertions that are not potentially harnessed by bacterial sequences derived from the plasmid backbone. A recent *in vivo *study has shown an increase in heterochromatin-like histone modifications correlating with lower levels of transgene expression from transfected plasmids containing bacterial sequences compared to plasmids devoid of bacteria-derived DNA [21]. Together, our findings provide a novel tool for Flp-based genetic engineering and pave the way for future applications of lentiviral DNA circles as potential substrates for more therapeutic relevant nonviral integration machineries in somatic gene transfer.

## Conclusion

Site-specific integration systems as Cre and Flp are important tools in genetic engineering and animal transgenesis but can in some instances be hampered by the requirement for transfection of plasmid DNA. Lentiviral vectors efficiently transduce both proliferating and quiescent cells but have a preference for integration into transcriptional units thereby representing a potential risk of insertional mutagenesis. A portion of the lentiviral DNA in transduced cells is converted into episomal circlular forms. We have herein demonstrated for the first time that an exogenous recombinase has access to lentiviral DNA as substrate for DNA insertion with the potential of altering the integration profile of HIV-1-derived vectors. In the present setup we combined lentiviral delivery with the site-specific integration properties of the Flp recombinase in a drug-selective approach. By packaging the viral vector with the inactive D64V integrase mutant we were able to increase the amount of circular substrates 4-fold and at the same time abolish the viral integration machinery. We demonstrate that both 1- and 2-LTR circles were inserted into engineered FRT acceptor sites in the genome of human cells by *trans*-acting Flp recombinase delivered either by Flp-encoding transfected plasmid DNA or by co-transduced integrase-defective lentiviral vectors carrying a Flp expression cassette. Among several advantages of this approach, genetic cargo inserted as viral DNA circles is not potentially harnessed by bacterial sequences derived from the plasmid backbone, the latter which is believed to negatively affect the long-term stability of transgene expression. Moreover, this hybrid lenti-Flp technology may be usefull in studies that require Flp-mediated gene insertion in hard-to-transfect cell lines. Our findings provide evidence that *trans*-acting integrases are able to gain access to and insert transduced lentiviral DNA and open up for the development of new hybrid systems which do not rely on an engineered genomic target sequence and which are based on more therapeutically relevant recombinases or transposases for potential use in gene transfer.

## Authors' contributions

BM, NHS, and JGM conceived and designed the experiments and the hybrid vector constructs. RJYM assisted during development of the experimental design. All vectors were constructed by BM. Cell culture analyses were performed by BM, and molecular analyses were carried out by BM, NHS and MJ. JGM mentored BM, NHS and MJ in construction work and data analysis. BM drafted the manuscript along with NHS and JGM, and JGM completed the manuscript preparation. All authors read and approved the final manuscript.
